# Impact of COVID-19 on the Dental Community: Part I before Vaccine (BV)

**DOI:** 10.3390/jcm10020288

**Published:** 2021-01-14

**Authors:** Sameh Attia, Hans-Peter Howaldt

**Affiliations:** Department of Oral and Maxillofacial Surgery, Justus-Liebig University of Giessen, Klinikstr. 33, 35392 Giessen, Germany; HP.Howaldt@uniklinikum-giessen.de

One year has passed with the COVID-19 pandemic and its impact is still evident everywhere on the globe and in all fields and domains. It was very clear that we all were not prepared for such a situation which was completely new for the living generation. However, the light of optimism has been shed with the onset of vaccination.

From beginning of the pandemic, authors realized the imminent impact on the dental field. The route of transmission via contact with droplets and aerosols puts dentists at high risk of infection. Dental treatment became a challenge during the outbreak. Initially, the publications dealing with the COVID-19 and the dental field were limited; then, we had a stream of papers with different quality grades. With this Special Issue, we aim to act as a scientific meeting point for all kinds of high-quality research dealing with the management of the COVID-19 crisis within the dental community. However, the main problem faced by scientific groups in multiple countries has been that health authorities were hesitant to approve clinical investigations for research purposes on COVID-19 patients to reduce the spread of infection.

To date, we have been able to publish eight high-quality research and review papers which significantly added knowledge to the scientific community and the recognized progress of the pandemic. With the beginning of vaccination, a very important milestone was achieved, and we recommend dividing the pandemic time into: before vaccine (BV) and after vaccine (AV). This classification allows a better understanding of the research circumstances, psychologies, and perspectives.

In the first pandemic phase (BV), most non-essential dentist visits were canceled or postponed to avoid the spread of infection. Tele-dentistry arose as a promising field, allowing to maintain recall visits without physical contact [1]. This technology can be also used post-COVID especially in some dental disciplines and when clinical or radiological investigations are not required. However, dental emergency treatment is still available and requires a physical visit to the dental office. While dental anxiety of children under pandemic circumstances did not show any increase, as it was tested in a specific group [2] another study presented an enhancement of dental anxiety among patients undergoing oral surgery procedures [3]. Providing treatment for oral cancer patients can be considered as an urgent treatment that has to be maintained during the pandemic. This goal can be achieved with an adequate treatment protocol to protect immunocompromised patients and involved health care professional workers [4]. Other protocols using bio-inspired systems in nonsurgical periodontal treatment led to a reduction in aerosol generation and therefore limited the spreading of COVID-19 infection [5]. Over time, it became clear that the SARS-CoV-2 virus directly affected the oral cavity with several signs and symptoms such as xerostomia, impaired taste, burning sensation, and difficulty in swallowing [6,7]. An indirect impact of Coronavirus was detected in patients in two countries who had worsening bruxism and temporomandibular dysfunction (TMD) symptoms [8].

It can be concluded from the present editorial which contains a summary of the published papers usque ad diem, that we learned a lot about the pandemic in its first phase (BV). There remains a lot of work to do in the next phase of the pandemic (AV). All scientific works and well-conducted clinical reviews regarding the COVID-19 outbreak and its effects on the dental field are more than welcome and of great importance in the second pandemic phase.

## References

[1] Maspero C., Abate A., Cavagnetto D., El Morsi M., Fama A., Farronato M. (2020). Available technologies, applications and benefits of teleorthodontics. A literature review and possible applications during the COVID-19 Pandemic. J. Clin. Med..

[2] Olszewska A., Rzymski P. (2020). Children’s Dental Anxiety during the COVID-19 Pandemic: Polish Experience. J. Clin. Med..

[3] Pylińska-Dąbrowska D., Starzyńska A., Cubała W.J., Ragin K., Alterio D., Jereczek-Fossa B.A. (2020). Psychological Functioning of Patients Undergoing Oral Surgery Procedures during the Regime Related with SARS-CoV-2 Pandemic. J. Clin. Med..

[4] Manuballa S., Abdelmaseh M., Tasgaonkar N., Frias V., Hess M., Crow H., Andreana S., Gupta V., Wooten K.E., Markiewicz M.R. (2020). Managing the Oral Health of Cancer Patients During the COVID-19 Pandemic: Perspective of a Dental Clinic in a Cancer Center. J. Clin. Med..

[5] Butera A., Maiorani C., Natoli V., Bruni A., Coscione C., Magliano G., Giacobbo G., Morelli A., Moressa S., Scribante A. (2020). Bio-Inspired Systems in Nonsurgical Periodontal Therapy to Reduce Contaminated Aerosol during COVID-19: A Comprehensive and Bibliometric Review. J. Clin. Med..

[6] Sinjari B., D’Ardes D., Santilli M., Rexhepi I., D’Addazio G., Di Carlo P., Chiacchiaretta P., Caputi S., Cipollone F. (2020). SARS-CoV-2 and Oral Manifestation: An Observational, Human Study. J. Clin. Med..

[7] Coke C.J.D.B., Fields N., Fletcher J., Rollings J., Roberson L., Challagundla K.B., Sampath C., Cade J., Farmer-Dixon C., Gangula P.R. (2021). SARS-CoV-2 Infection and Oral Health: Therapeutic Opportunities and Challenges. J. Clin. Med..

[8] Emodi-Perlman A., Eli I., Smardz J., Uziel N., Wieckiewicz G., Gilon E., Grychowska N., Wieckiewicz M. (2020). Temporomandibular Disorders and Bruxism Outbreak as a Possible Factor of Orofacial Pain Worsening during the COVID-19 Pandemic—Concomitant Research in Two Countries. J. Clin. Med..

